# Adaptive tutorials versus web-based resources in radiology: a mixed methods analysis in junior doctors of efficacy and engagement

**DOI:** 10.1186/s12909-020-02237-8

**Published:** 2020-09-14

**Authors:** Stuart W. T. Wade, Michelle Moscova, Nicodemus Tedla, Daniel A. Moses, Noel Young, Merribel Kyaw, Gary M. Velan

**Affiliations:** 1 Prince of Wales Hospital, Sydney, Australia; 2 School of Medical Sciences, Faculty of Medicine, The University of New South Wales, Sydney, Australia; 3 Prince of Wales Clinical School, Faculty of Medicine, The University of New South Wales, Sydney, Australia; 4 Westmead Hospital, Sydney, Australia; 5 Royal Prince Alfred Hospital, Sydney, Australia

**Keywords:** Radiology, Diagnostic imaging, Medical education, Adaptive tutorials, Junior doctors

## Abstract

**Background:**

Radiology education is limited in undergraduate Medicine programs. Junior doctors might not have the necessary background to effectively order and interpret diagnostic imaging investigations. Furthermore, junior doctors are often time-poor, balancing clinical commitments with ongoing learning, leadership and teaching responsibilities. Previous studies have demonstrated the efficacy of radiology-themed online adaptive tutorials for senior medical students. Such adaptive tutorials might therefore be an efficient and effective form of radiology education for junior doctors.

**Methods:**

A randomised controlled crossover trial was performed to evaluate the impact of adaptive tutorials on learning the indications for, and interpretation of, basic imaging studies, compared with peer-reviewed web-based resources. Ninety-one volunteer junior doctors, comprising 53 postgraduate year 1 (PGY 1) and 38 postgraduate year 2 (PGY 2), were randomly allocated into two groups. In the first phase of the trial, focusing on head CT, one group accessed adaptive tutorials while the other received web-based resources. In the second phase of the trial, focusing on chest CT, the groups crossed over. Following each phase of the trial, participants completed exam-style online assessments. At the conclusion of the study, participants also completed an online questionnaire regarding perceived engagement and efficacy of each type of educational resource.

**Results:**

Junior doctors completed the adaptive tutorials significantly faster than the relevant web-based resources for both head CT and chest CT (*p* = 0.03 and < 0.01 respectively). Mean quiz scores were higher in the groups receiving adaptive tutorials on head CT and chest CT (86.4% vs 83.5 and 77.7% vs 75% respectively). However, in contrast to previous studies in senior medical students, these differences were not statistically significant. Participants reported higher engagement and perceived value of adaptive tutorials, compared with web-based resources.

**Conclusions:**

Adaptive tutorials are more time-efficient than existing web-based resources for learning radiology by junior doctors, while both types of resources were equally effective for learning in this cohort. Junior doctors found the adaptive tutorials more engaging and were more likely to recommend these resources to their colleagues.

## Background

Radiology education is limited in medical school programs worldwide, in which there are often no standardized radiology curricula [1–6]. For junior (pre-vocational) doctors, the potential effects of this lack of education in radiology include over utilisation of imaging resources, limited awareness of risks such as radiation exposure and impaired ability to successfully interpret basic imaging [5–11]. Diekhoff and colleagues reported that tutorials for medical students on imaging indications resulted in significantly more appropriate medical imaging examinations being selected for individual case scenarios [12].

There are limited opportunities for junior doctors to engage with radiology education [13]. Junior doctors are often time-poor, balancing clinical responsibilities with ongoing learning which is often geared toward their desired future specialty. Many also engage in extracurricular activities, such as mentoring of medical students, teaching, research and leadership positions aimed at furthering their career prospects. Many hospitals have structured teaching programs with protected face to face teaching time. However, attendance can be variable. Moreover, few places exist for radiology rotations for pre-vocational junior doctors [13]. Thus, online learning (e-Learning) might be useful to assist the continuing development of junior doctors by providing a platform unconstrained by time and space.

Radiology education is particularly suited to e-Learning, as most diagnostic imaging is currently digitised. The use of Picture Archiving and Communication Systems (PACS) at many hospitals has resulted in a wealth of easily accessible images. With appropriate institutional approval, such images can be anonymised, exported and integrated into e-Learning modules in radiology. Many freely available online resources already utilise PACS images and are anecdotally reported to be popular among junior doctors. While these resources can be effective in filling the knowledge gap, they are mostly focused on passive information delivery and do not utilise best principles of online instructional design. Customised resources designed using multimedia learning principles to provide learners with guided instruction may be more effective, but they are also more expensive to produce. In addition, for junior doctors, there are few studies that compare the effectiveness of customised resources with freely available web-based resources.

Adaptive tutorials are a form of online intelligent tutoring system [14–16], enabling asynchronous e-Learning. These tutorials provide guided instruction with immediate personalised feedback on the user’s input.

The guided instruction provided by adaptive tutorials is in keeping with the best principles of educational design, particularly multimedia learning principles and cognitive load theory. This theory addresses how learners have limited cognitive capacity for processing information in their working memory which, if exceeded, may result in cognitive overload [17, 18]. The result of cognitive overload is incomplete or disorganised understanding, which may result in misconceptions [17, 18]. Cognitive overload may occur with unguided learning, excessive information or resources that are not tailored to a particular audience’s level of understanding [17, 18]. To avoid cognitive overload, the depth of information in the adaptive tutorials is aimed specifically at a senior medical student / junior doctor level of understanding (or stage of training). In addition, the material is segmented or ‘chunked’ into manageable portions or more complex concepts introduced incrementally with examples and feedback.

Previous controlled crossover studies by Wong et al. [14] and Wade et al. [15] demonstrated that adaptive tutorials were effective tools for radiology education, and were well-accepted by junior and senior medical students respectively. In particular, medical students favoured the engaging, interactive nature of the adaptive tutorials when compared with peer reviewed web-based resources Radiopaedia (https://radiopaedia.org/) and Diagnostic Imaging Pathways (http://www.imagingpathways.health.wa.gov.au/) [14, 15]. This was reflected in objective measures of learning, whereby junior medical students who received adaptive tutorials displayed significantly better knowledge of indications and interpretations of basic imaging in online quizzes than those students exposed to equivalent peer-reviewed web-based resources [14, 15].

The efficacy of adaptive tutorials compared with web-based resources was less clear-cut for senior medical students [15]. While the groups in both arms of the study receiving adaptive tutorials achieved higher mean quiz scores than those receiving peer-reviewed web-based resources, the difference was statistically significant only in the first phase of the study, focusing on CT imaging of the head [15].

Building on previous studies, we utilised a randomised crossover trial to evaluate the educational impact of radiology-themed adaptive tutorials targeting junior doctors, compared with peer-reviewed web-based resources. The adaptive tutorials, web-based resources and associated quizzes were identical to those utilised in the previous study undertaken with senior medical students [15].

## Methods

The educational resources, quizzes, surveys and methods of analysis in the present study were identical to those described in a recent study of senior medical students by Wade et al. [15]. Unlike the previous study, the participants in this study consisted of junior doctors in their first and second years of pre-vocational supervised practice following graduation. Approval for this study was obtained from UNSW Human Research Ethics Committee (HC17939). The materials and methods are briefly described below.

### Development and organisation of educational materials

Adaptive tutorials covering computed tomography (CT) scans of the head and chest were developed using Smart Sparrow’s (http://smartsparrow.com) Adaptive e-Learning Platform (AeLP). These tutorials were aimed at the level of senior medical students and prevocational junior doctors. Each module was divided into three sections:

1. Introduction and indications for common imaging investigations and a suggested checklist for basic image interpretation.
2. Commonly encountered clinical scenarios where participants were asked to select the most appropriate imaging investigations. Participants were asked to interpret the imaging by identifying anatomical and pathological features.
3. Estimated ionising radiation dosages of many commonly encountered imaging investigations and a comparison made with naturally occurring background radiation.

**Fig. 1:**
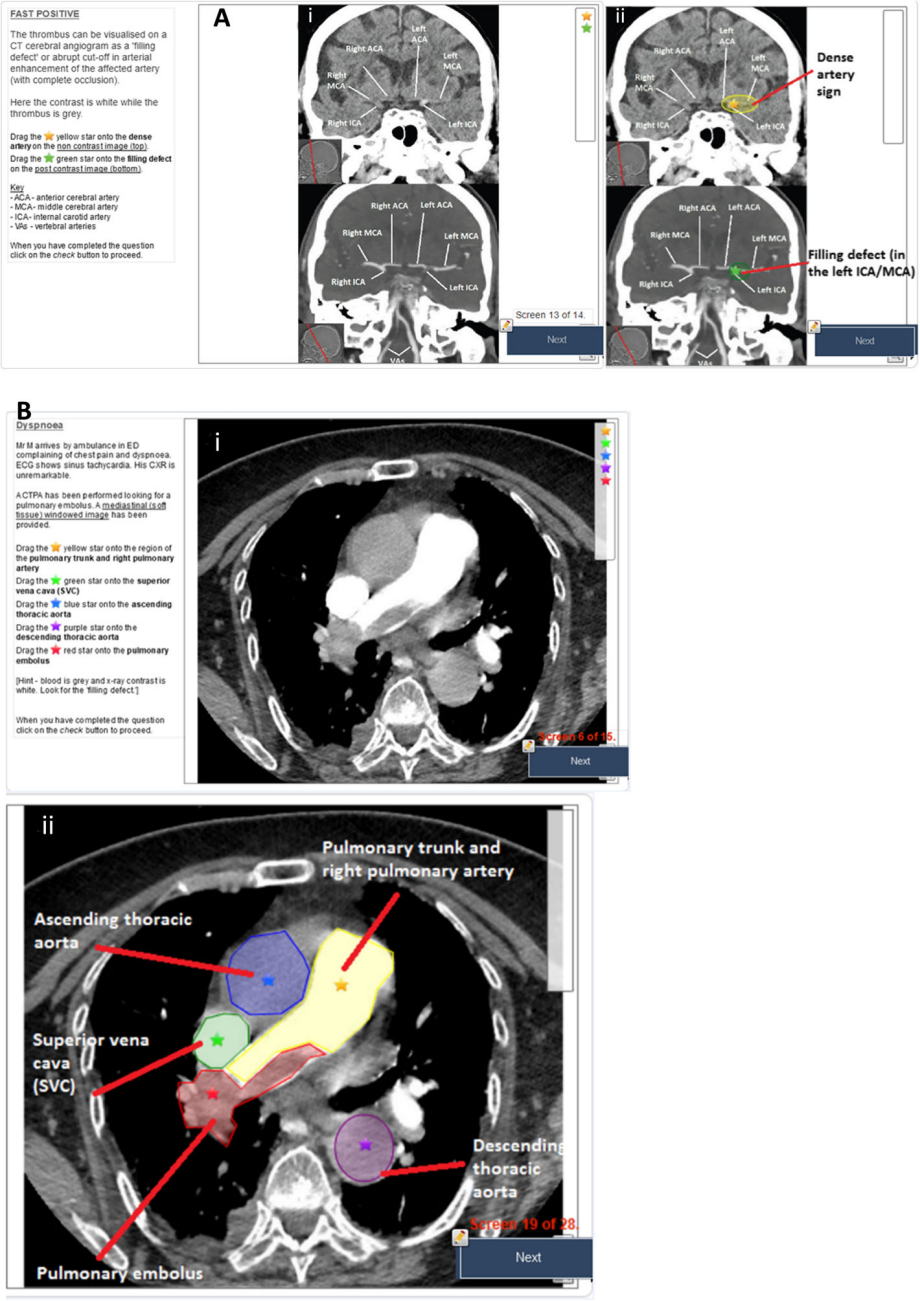
Representative examples of drag and drop interaction in adaptive tutorials on CT scans of the head (**a**) and chest (**b**), with feedback following submission of answers

Interactions in the adaptive tutorials consisted of multiple-choice questions, drop down lists and drag and drop labelling identification of specific features on imaging (Fig. 1a and b). Immediate personalised feedback was provided after each response. These tutorials are modelled to encourage psychological engagement and resultant knowledge construction. Clinical scenarios are presented and participants encouraged to identify relevant clinical information, produce hypotheses in the context of their medical experience and provisional diagnoses. The information is provided in didactic format and as part of clinical scenarios either directly or as feedback. Unlike many available resources which are not aimed at a particular audience, the pre-requisite knowledge, information presented and language used is aimed at senior medical students and junior doctors. The information is presented in a concise, structured format as small manageable portions to prevent cognitive overload and possible confusion or generation of ‘misconceptions.’

The control group was provided with links to existing web-based articles available from Radiopaedia and Diagnostic Imaging Pathways, both peer reviewed resources. Radiopaedia is an open edit, online radiology resource consisting of radiology related information articles and radiology case studies compiled by radiologists and other health professionals worldwide. Diagnostic imaging pathways provide an evidence-based decision support tool for clinicians in a wide range of clinical scenarios.

Smart Sparrow’s Adaptive eLearning Platform (AeLP) enables accurate recording of time taken to interact with educational resources. Hence, participants accessed web-based resources through web-based materials embedded within I-Frames on the AeLP to allow accurate measurement of time spent engaging with these materials. No interactive features available on the AeLP were utilised for the web-based resources, thus mirroring the experience of accessing these resources independently via the web.

Hyperlinks to the adaptive tutorials, peer-reviewed web-based resources and quizzes were individually emailed to each participant’s during each phase of the study. After clicking on the link, the participants accessed the relevant learning activity or quiz.

### Development of online assessments of knowledge

Online assessments were developed using Questionmark Perception (Questionmark Computing Ltd., London, UK). Three assessments were developed comprising a baseline quiz and quizzes on imaging of the head and chest to be completed at the end of each phase of the study. These were identical assessments to those used in the previous study in senior medical students [15]. Each assessment had a 15-min time limit and automated feedback was provided at the conclusion of each assessment.

### Development of online questionnaires regarding perceptions of engagement and utility

An online questionnaire based on the Perceived Utility of Learning Technologies Scale (PULTS – Additional file 1) was employed to gather students’ quantitative and qualitative perceptions of both Adaptive Tutorials and Web-Based resources. PULTS was developed and internally validated at our institution and has been utilised in previous studies [15, 16].

To prevent bias, educational resources and tests were reviewed by the research team, three of whom were not radiology trained. The participants in the adaptive tutorials and web-based resources groups were exposed to identical topic areas in each phase of the study.

### Participants

Intern (postgraduate year 1 - PGY 1) and resident (postgraduate year 2 - PGY 2) doctors employed in hospitals by New South Wales Health were eligible for inclusion in the study. The definition of junior doctors varies and for the purposes of this study, junior doctors are prevocational doctors who were yet to specialise and were undertaking rotations in various medical, surgical and critical care units. More senior doctors beyond PGY 2 and those in specialty training were excluded from the study. Volunteer participants were recruited by broadcast email, with support of the NSW Health Education and Training Institute (HETI). Recruitment and data collection were performed annually over two consecutive years (2018 and 2019).

A total of 91 junior doctors volunteered for the study, consisting of 53 PGY 1 and 38 PGY 2 doctors. During the 2019 recruitment, participants were screened to ensure they had not previously participated in this study. A power analysis determined that a minimum of 18 participants per study group was required to detect a 20% difference between groups with 99% statistical power.

### Study design

A randomised crossover control trial was performed. Prior to commencement, participants completed an online quiz to ascertain their baseline knowledge of diagnostic imaging, then were randomized into two groups stratified by years of postgraduate experience (PGY 1 and PGY 2). Each phase of the trial was of one-week duration, followed by an examination-style online quiz on the topic covered by the educational resources provided in that phase. The first phase of the trial focused on CT scans of the head, while the second phase covered CT scans of the chest. In the first phase, one group received adaptive tutorials and the other group accessed web-based resources. Following the crossover, the group that had previously been exposed to peer-reviewed web-based resources accessed adaptive tutorials, and vice versa. After completing the final quiz, participants were asked to respond to an online exit survey (PULTS). This timeline is summarised in Table 1.

**Table 1:** Timeline and format of the radiology adaptive tutorials trial

Topic	Group A	Group B	Timeline (Days)
	Baseline Online Quiz		0-7
**CT Head**	Adaptive Tutorials	Web-Based Resources	8-14
	Online Quiz on CT of the Head		15-21
**CT Chest**	Web-based Resources	Adaptive Tutorials	22-28
	Online Quiz on CT of the Chest and Online Questionnaire		29-34

### Statistical analysis

Statistical analysis was performed using GraphPad Prism version 7.04 for Windows (GraphPad Software, La Jolla California USA, www.graphpad.com). Unpaired Student t-tests were performed to evaluate for differences in mean scores between groups per assessment, as well as for differences in time taken to complete each type of learning resource. For those analyses, data are presented as means with 95% confidence intervals unless otherwise stated.

Analysis of online questionnaire data for differences in participants’ perceptions of adaptive tutorials and web-based resources was performed using a Mann-Whitney rank-sum test for unpaired variables and a Wilcoxon rank sign test for paired variables. Qualitative analysis of open-ended questionnaire responses was performed using Dedoose version 8.0.42, a web application for managing, analysing and presenting qualitative and mixed method research data (Los Angeles CA: Socio-Cultural Research Consultants, LLC - www.dedoose.com). Responses to four open-ended questionnaire items assessing positive aspects and recommended improvements for the adaptive tutorials and web-based resources were exported into Dedoose to enable qualitative analysis. Two researchers reviewed the data and performed initial coding independently (SW and MM). The data was coded using open coding. The codes were subsequently discussed by both researchers to achieve consensus. Common themes were then identified until inductive thematic saturation was obtained [19]. Data were categorized according to the emergent themes and relationships between themes and categories were identified.

## Results

### Baseline test and randomisation of participants

A total of 91 volunteer participants attempted the baseline quiz, consisting of 53 PGY 1 and 38 PGY 2 doctors. The average score was 72.7% (95% CI = ± 3.2%) with a maximum score of 100 and a minimum score of 33. When analysed according to level of training, mean scores for PGY 1 and PGY 2 participants in the baseline quiz were 73.5% (95% CI = ± 4%) and 71.6% (95% CI = ± 5.5%) respectively. As there was no statistically significant difference in quiz scores according to seniority of participants (*p* = 0.56), all subsequent analyses utilised pooled results from PGY 1 and PGY 2 doctors in each group.

**Fig. 2:**
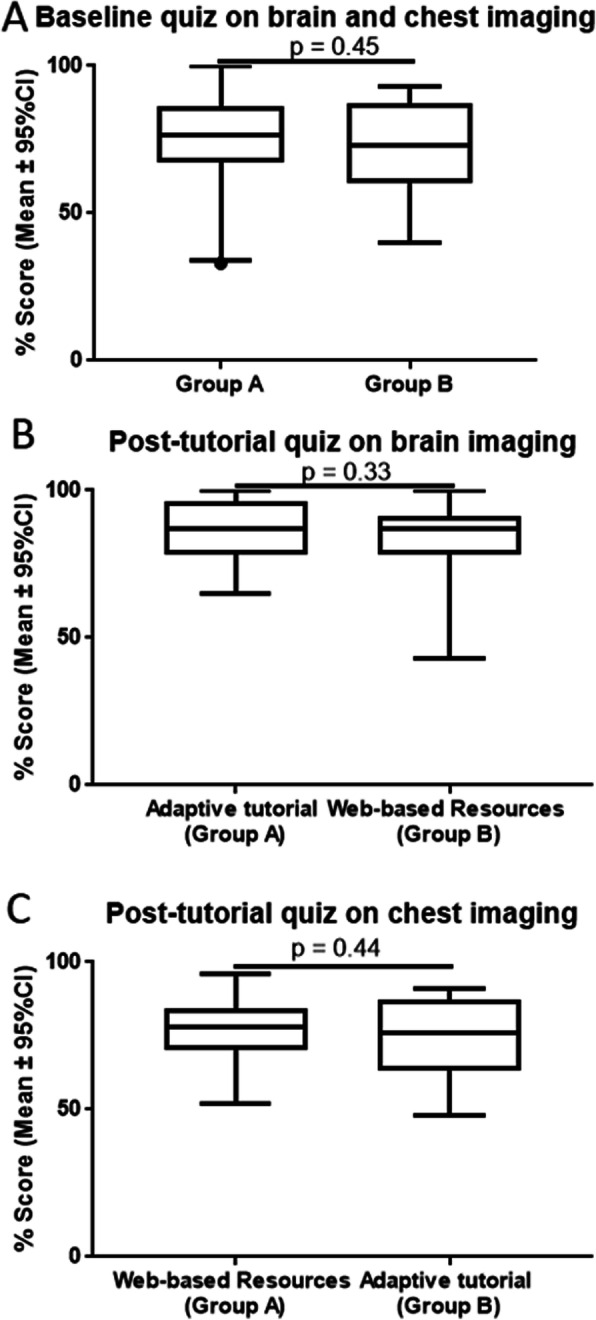
Baseline imaging quiz scores for both groups (**a**), quiz scores for the head CT quiz for phase 1 of the study (**b**) and the chest CT quiz for phase 2 of the study (**c**). (Mean with 95% CI and range)

Participants were subsequently randomised into two groups, stratified by year of training. Group A consisted of 44 participants (26 PGY1 and 18 PGY2) with a mean baseline quiz score of 74% (95% CI = ± 4.8%) and group B consisted of 47 participants (27 PGY1 and 20 PGY2) with a mean score of 71.51% (95% CI = ± 4.4%). There was no statistically significant difference in baseline quiz scores between groups (*p* = 0.45) (Fig. 2a).

### Phase 1 – CT of the head

In phase 1 of the study, group A received the adaptive tutorials and group B received the web-based resources on CT scans of the head. A total of 62 participants completed phase 1 of the trial. Thirty participants in group A completed the quiz, with a mean score of 86.4% (95% CI = ± 3.8%; range 65–100%). Thirty-two participants in group B completed the quiz, with a mean score of 83.5% (95% CI = ± 4.7%; range 43–100%). There was no statistically significant difference in mean quiz scores between groups (*p* = 0.33) (Fig. 2b).

### Phase 2 – CT of the chest

In phase 2 of the study, group A received web-based resources and Group B received the adaptive tutorials on CT scans of the chest. A total of 50 participants completed phase 2 of the study. Twenty-six participants in group A completed the quiz, with a mean score of 77.7% (95% CI = ± 4.6%; range 52–96%). Twenty-four participants in group B completed the quiz with a mean score of 75% (95% CI = ± 5.7%; range 48–91%). Again, there was no statistically significant difference in mean quiz scores between groups (*p* = 0.44) (Fig. 2c).

### Time spent using educational resources

In both phases of the study, adaptive tutorials were completed in a shorter average time than web-based resources. The differences were statistically significant in both phases of the study, as summarised in Table 2.

**Table 2:** Time Spent using Educational Resources

	Adaptive Tutorial	Web-Based Resources	***P***-value
**Head CT**	31 min 30 s (95% CI = ± 12 min 4 s)	70 min 54 s (95% CI = ± 35 min 22 s)	0.03
**Chest CT**	20 min and 59 s (95% CI = ± 9 min 10 s)	59 min 47 s (95% CI = ± 21 min 50 s)	< 0.01

### Outcomes of online questionnaires

**Fig. 3:**
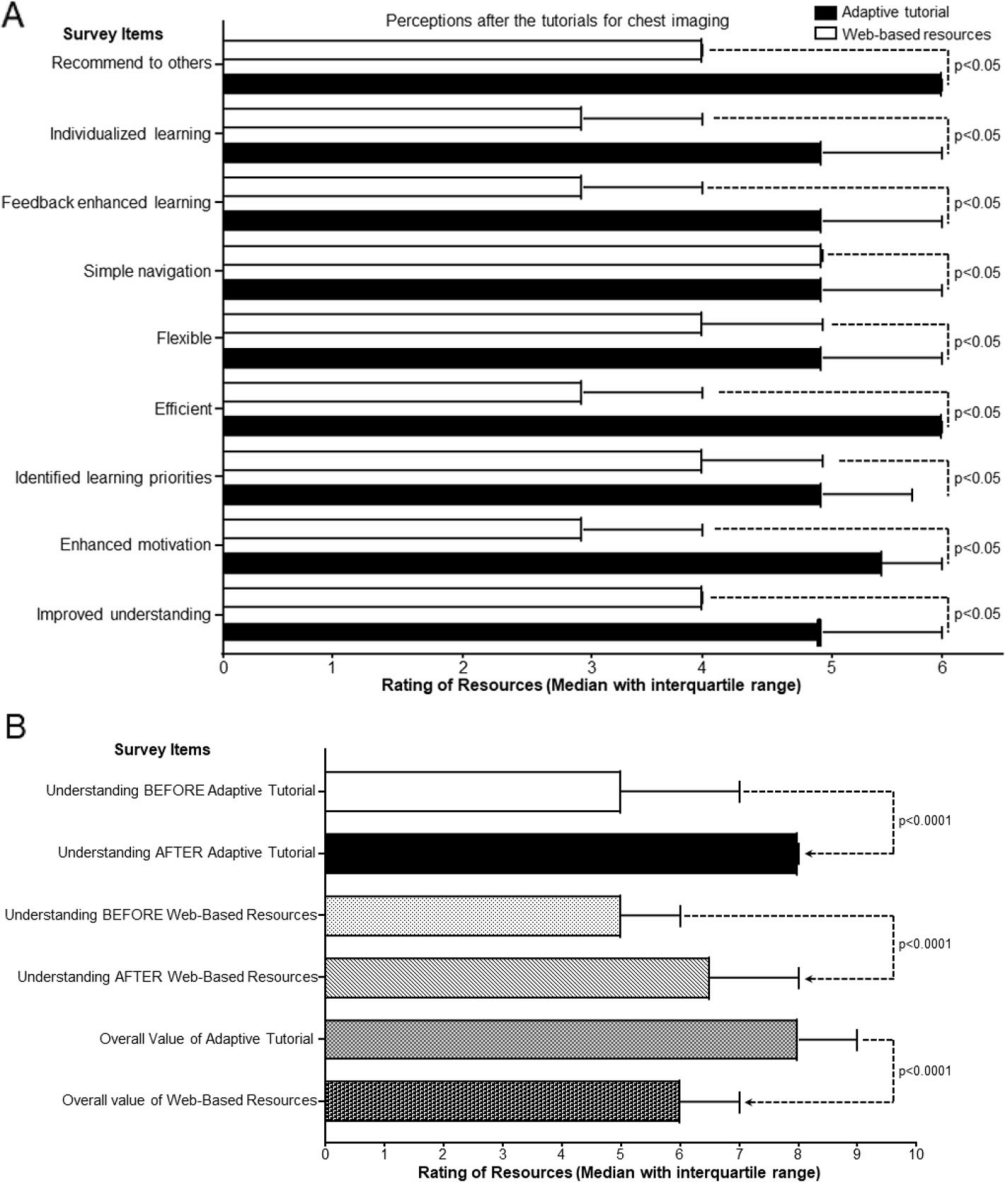
**a**: Exit questionnaire analysis of the perceived utility of adaptive tutorials compared with web-based resources (median score with an interquartile range; *p ≤ 0.05, Mann Whitney). Likert scale – 1 strongly disagree to 6 strongly agree. **b**: Perceived improvement in understanding and overall perceived value of adaptive tutorials compared with web-based resources (Median score with an interquartile range; *p ≤ 0.0001, Wilcoxon Ranked Sign test). Likert scale – 0 least understanding to 10 most understanding. Likert scale – 0 not useful to 10 extremely useful

Survey results from the 40 participants who completed the online questionnaires revealed statistically significant differences in perceptions of utility in favour of adaptive tutorials across all items (*p* ≤ 0.05). Interestingly, junior doctors perceived that both adaptive tutorials and web-based resources positively impacted their understanding of the topic areas assessed. However, participants rated the adaptive tutorials significantly higher regarding their overall value as learning tools (*p* < 0.0001). A summary of the questionnaire results is shown in Fig. 3.

### Qualitative analysis

**Fig. 4:**
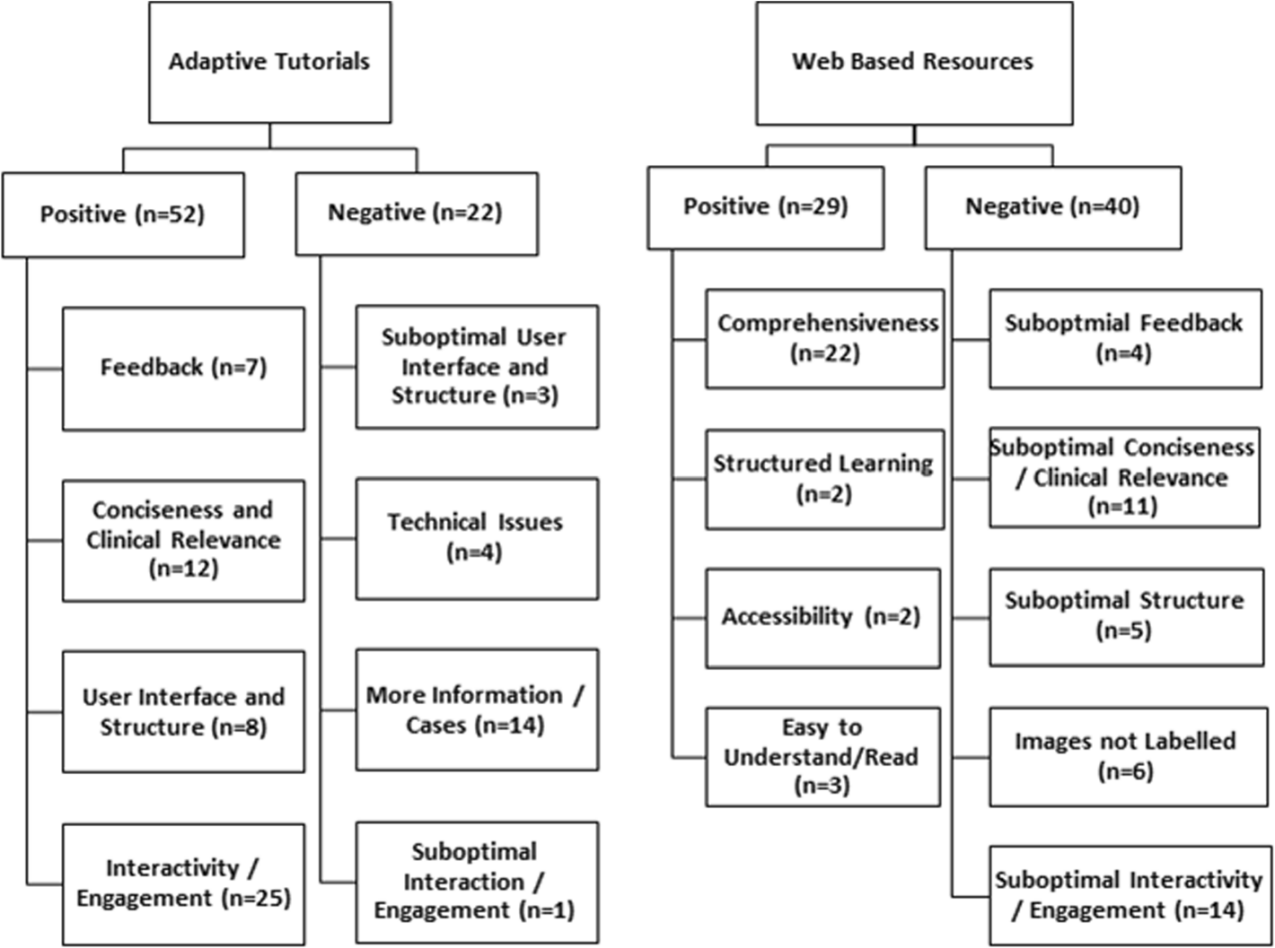
Major qualitative themes derived from open-ended feedback regarding adaptive tutorials (**a**) and web-based resources (**b**)

Themes derived from open-ended questionnaire responses regarding each type of educational resource are summarised in Fig. 4.

#### Adaptive tutorials

The most common theme for favourable comments on the adaptive tutorials was ‘interactivity and engagement’ (*n* = 25). Two main subthemes emerged where junior doctors appreciated the question-based nature of the tutorials (*n* = 9) and drag and drop questions (*n* = 4). Interestingly PGY 1 doctors listed more favourable comments then PGY 2 doctors (21 versus 4). ‘Conciseness and clinical relevance’ was the next most common positive theme (*n* = 12) followed by ‘user interface and structure,’ (*n* = 8). From the ‘user interface and structure,’ many participants enjoyed the ‘case-based structure’ which was based on common clinical scenarios. Participants also commented on adaptive tutorials providing a ‘clear diagnostic approach’ and ‘guided study.’ Feedback again was a prevalent theme (*n* = 7).

The most cited area for improvement in the adaptive tutorials was a request for ‘more information / cases,’ (*n* = 14). Among those responses, two participants asked for more images / examples, 6 for more information and 4 for more questions. Overall, more PGY 1 than PGY 2 junior doctors requested more information (12 versus 2). Technical issues were the second most cited area for improvement (*n* = 4), mainly consisting of browser issues or loading of drag and drop questions. From the ‘suboptimal user interface theme,’ (*n* = 3), two PGY 2 doctors requested a facility to revisit previous screens and one PGY 2 doctor asked for stacks of cross-sectional images to be displayed rather than selected single images.

#### Web-based resources

Junior doctors overwhelmingly perceived the web-based resources to be more comprehensive than the adaptive tutorials (*n* = 22). Two respondents noted the wide range of examples and 1 respondent appreciated the links to further resources. Otherwise junior doctors felt the resources were easy to understand or read (n = 3).

However, junior doctors perceived that web-based resources provided suboptimal interactivity or engagement (*n* = 14), particularly PGY 1 doctors (*n* = 13). Of that group, 4 would have preferred more test questions or more clinical cases. The second most cited area for improvement was suboptimal concision of information or lack of clinical relevance (*n* = 11). Otherwise, 6 respondents requested labelling of images, 5 wished for better presentation of information and 4 asked for addition of questions and feedback. A set of responses best summarising the key themes are detailed in Table 3.

**Table 3:** Representative selection of key themes from the open-ended questionnaire responses

Educational Resource	Response	Key Themes Addressed^a^
**Adaptive Tutorials**	“It was very efficient way to learn, it was fast but really made sense and increased my understanding of the topic. It also made it enjoyable, less of a task to be done and something that actually felt satisfying to complete and useful. I have already found myself more confident assessing head imaging I order as a result. I liked the way it [uses] simple language to describe what I’m seeing and what I need to look for. I also liked the way it walked us through a clinical case rather than just talking about it in abstract.”	IAE, FEE
	“Interactive and efficient learning time! I particularly liked the “drop and drag” tools and colour coding anatomy.”	IAE, CCR
	“More options to spend longer on weaker areas - links for further reading/learning.”	MIC
	“It is difficult to assess the imaging solely based on a static picture. It would be easier if you could scroll as you normally do to better assess the anatomy and abnormalities.”	SUS
**Web-based** **Resources**	“Huge amount of well written information. Need to be committed to reading such a large amount though. Some explanations and flow provided better instruction for differentiation and what to expect on imaging than the adaptive tutorial.”	COM, SIE
	“Systematic approach with good overview and great example images.”	COM, STL
	“Diversity of topics, self-guided, fast access when you are just hoping to look up one particular thing.”	COM, ACC
	“Hard to follow, not interesting or engaging. The way the images do not directly follow or relate to the text makes it hard to see what I’m supposed to be seeing.”	SIE, INL
	“Too much information sometimes needs more guidance in the example images - e.g. point out/highlight the abnormality.”	SCC, SST

^a^*IAE* Interactive and engaging, *FEE* Feedback, *CCR* Conciseness and clinical relevance, *MIC* More information and / or cases, *SUS* Suboptimal user interface and structure, *COM* Comprehensiveness, *SIE* Suboptimal interactivity and engagement, *STL* Structured learning, *ACC* Accessibility, *INL* Images not labelled, *SCC* Suboptimal conciseness or clinical relevance, *SST* Suboptimal structure

## Discussion

In contrast to our previous investigations in junior [14] and senior medical students [15], there were no statistically significant differences in mean quiz scores between groups in both phases of the present study. This may be related to junior doctors’ greater experience and clinical exposure, compared with medical students. The added experience of junior doctors might have resulted in higher baseline understanding, as evidenced by the high baseline quiz scores. Moreover, experienced learners do not only possess greater knowledge, they are also better at organising and retrieving relevant information required for problem-solving [20]. Clinical expertise develops gradually, over the years in response to progressive problem-solving with gradual increase in complexity of clinical problems [20]. Novice learners are more dependent on instructor guidance, scaffolding of problems and instructor assistance to identify relevant information for solving a particular problem [20]. More experienced learners are able to connect information from multiple sources and are more independent in seeking further feedback and knowledge [20]. This might have enabled junior doctors to more effectively utilise web-based resources compared to medical students. Nevertheless, adaptive tutorials took significantly less time to complete than web-based resources in both phases of the study. Therefore, although adaptive tutorials and web-based resources were equally effective in enhancing quiz performance, adaptive tutorials achieved their benefits more efficiently. As junior doctors are time-poor, brevity of the learning activity may act as an extra incentive for them to complete it.

Junior doctors found the adaptive tutorials to be more interactive and engaging than web-based resources. This might have affected participation rates between phases of the study. Group A, which received adaptive tutorials in the first phase, had a drop-out rate of 13% (4 of 30). In contrast, group B, which accessed web-based resources in the first phase, had a drop-out rate of 25% (8 of 32). In particular, the ‘drag and drop’ questions in adaptive tutorials were appreciated by both groups, wherein participants were asked to identify abnormalities on diagnostic images. This task is similar to what junior doctors are asked to do routinely in daily practice and is consistent with the concept of ‘mirroring’, which has been shown to improve future performance [21]. Junior doctors commented favourably on the utility of feedback provided by the adaptive tutorials.

According to Clark and Mayer, engagement may be behavioural or psychological [21]. Behavioural engagement varies from pressing a ‘next button’ to selecting options from multiple choice of drop-down menus [21]. On the other hand, adaptive tutorials aim to maximise psychological engagement, i.e. making sense of information and applying it. Within adaptive tutorials, participants are encouraged to identify relevant material in the clinical scenario, integrating it with background medical knowledge and organising a hypothesis or provisional diagnosis. Participants are then asked to test this hypothesis by requesting relevant imaging and to interpret the imaging accordingly. In essence, adaptive tutorials encouraged ‘knowledge construction’ or maximising psychological engagement. By emphasising information likely relevant to junior doctors thus using the “signalling” principle [18], adaptive tutorials were perceived to be more concise and clinically relevant by junior doctors.

In contrast, many forms of online education such as the peer-reviewed web-based resources utilised in this study facilitate ‘information acquisition.’ This encourages the participant to be a passive recipient of information. Clark and Mayer [21] describe how some forms of behavioural engagement may depress relevant psychological activity or engagement and thus impede learning.

The volume of information presented in online educational resources requires a trade-off between attempts to be comprehensive and avoidance of cognitive overload. The goal is to present information in a manageable and meaningful way without overloading working memory of participants, as previously discussed [17, 18]. The guided instruction provided by adaptive tutorials aims at maximising ‘essential processing’ by presenting the most relevant material and segmenting the information into smaller manageable portions [21]. In addition, ‘generative processing’ or ‘knowledge construction’ is encouraged by the structure of adaptive tutorials. This involves fostering a deeper understanding of the material by promoting psychological engagement [21] and application of the material in clinical scenarios.

Thus, to avoid cognitive overload, the depth of information presented to participants in adaptive tutorials was limited to promote generative processing and deep learning. In contrast, given the didactic nature of the peer-reviewed web-based resources utilized in this study, there were less opportunities for generative processing, application and thus, deep learning. However, the breadth of information provided by web-based resources was greater.

The potential benefits of structuring adaptive tutorials via a case-based approach are two-fold. Firstly, a particular scenario may provide guidance or a ‘worked example’ of how to approach a complex clinical scenario, with individualized feedback. Worked examples are a powerful method to build complex cognitive skills by providing a guided demonstration of how to perform a task or solve a complex problem [21]. These worked examples may later be applied in the context of the participants’ background medical knowledge to similar scenarios in the future.

Overall, we found that ‘worked examples’ provided by adaptive tutorials had the greatest effect on learning of radiology by junior medical students [14] and to a lesser degree by senior medical students [15]. This may represent the ‘expertise reversal effect’ where worked examples may impede learning in those with greater expertise [19, 22]. Possible solutions for more experienced learners might include ‘worked examples’ which gradually fade into ‘practice questions,’ or to replace ‘worked examples’ with ‘practice questions’ in junior doctors.

The second potential benefit of the scenario-based nature of adaptive tutorials is that this may assist retrieval of learnt information from long term memory [21]. For example, a set of signs and symptoms or constellation of imaging findings in a scenario may serve as ‘retrieval hooks’ to ‘encode’ newly acquired information for later retrieval. This study did not evaluate learn term retention of educational materials. However, this might be an important area for future research.

A recurring theme in the exit questionnaire response was a request for labelling of images in the web-based resources [15]. Unlabelled images were felt to limit the educational utility of the web-based resources as participants did not always understand the anatomy or the pathology demonstrated by the images. This might also have negatively impacted the perceived engagement with web-based resources and could have contributed to the reduced participation rates in the second phase of the study. It is noted that group B, which received the web-based resources in the first phase of the study had a higher drop-out rate compared with group A, which received adaptive tutorials in the first phase. The increased dropout rate in the group receiving the web-based resources in the first phase of the study was also noted in our previous study in senior medical students [15]. This was exemplified by the statement in Table 3: “Too much information sometimes needs more guidance in the example images – e.g. point out / highlight the abnormality.” This suggests some peer-reviewed web-based radiology resources might be pitched at a level above the understanding of junior doctors, thus limiting their effectiveness. The confusion caused by unlabelled images might also partly explain why participants in the present study spent significantly longer using web-based resources than adaptive tutorials, without any incremental learning benefit.

The overall themes regarding perceptions of the learning experience were similar in both PGY 1 and PGY 2 doctors. A greater proportion of PGY 1 doctors commented on interactivity and engagement, volume and conciseness of information across both educational resources. Interestingly, PGY 2 doctors also requested a reverse function on the adaptive tutorials to re-review information and a stacking function to view cross-sectional images. Furthermore, a greater proportion of PGY 2 doctors favoured the case-based nature of the adaptive tutorials. These additional comments by PGY 2 doctors may reflect an element of pragmatism consistent with increased clinical experience.

### Limitations

The small size of the cohort of junior doctors recruited for this study might have limited the possibility of demonstrating significant differences between groups. Nevertheless, the study was adequately powered for this purpose. Furthermore, given the near identical quiz scores in both groups, it is unlikely that larger group sizes would have resulted in significant differences between mean quiz scores.

Participants’ quiz and survey results were anonymised, thus scores of those who did not complete all aspects of the study were included in the results. There was a greater drop out rate in the group receiving web-based resources (*n* = 8) versus the group which received the adaptive tutorials (*n* = 4) between phases of the trial. Whilst this supports the notion of greater perceived engagement with adaptive tutorials, there is a risk that this phenomenon might have skewed the results.

A consistent limitation of the adaptive tutorials utilised in this study was the inability to ‘scroll through’ stacks of cross-sectional images [15] which may queue participants to imaging findings. In reality, subtle pathologies such as segmental or subsegmental pulmonary emboli may only be visible on as little as 1 or 2 images in a stack. While stack viewing was enabled in web-based resources, the absence of labels limited their educational utility among non-radiology trained junior doctors.

## Conclusions

For junior doctors, interactive adaptive tutorials with automated feedback are a more time-efficient means of learning radiology, compared with peer-reviewed web-based resources. However, in this cohort of junior doctors, unlike junior and senior medical students, both types of educational resources were equally effective for learning. This finding provides helpful implications for resourcing of radiology education in junior doctors, who have the experience to derive more benefit than medical students from existing web-based resources. One option to potentially maximise the utility of adaptive tutorials in junior doctors might be to reduce the number of ‘worked examples’ and increase the number of practice exercises. Nevertheless, adaptive tutorials were preferred by junior doctors due to their interactive, engaging qualities, which closely mirror the tasks that junior doctors perform in their daily clinical practice. This might increase junior doctors’ motivation to complete adaptive tutorials.

Further studies are warranted of adaptive tutorials for radiology education in junior doctors, focusing on optimising the instructional design and evaluating both short-term and long-term learning gains. Additionally, studies comparing the learning benefits of high-fidelity (three-dimensional images stacks) with low-fidelity online educational resources in radiology might be of value.

## Supplementary information

**Additional file 1.** Perceived Utility of Learning Scale (PULTS). This is an online questionnaire to gather participants quantitative and qualitative perceptions of adaptive tutorials and web-based educational resources in radiology.

## Data Availability

The datasets used and/or analysed during the current study are available from the corresponding author on reasonable request.

## Abbreviations

PGY: Post graduate year
CT: Computed tomography
PACS: Picture Archiving and Communication System
AeLP: Adaptive e-Learning Platform
PULTS: Perceived Utility of Learning Technologies Scale
NSW: New South Wales
HETI: Health Education and Training Institute
