# Supplementary material for: Adaptive tutorials versus web-based resources in radiology: a mixed methods analysis in junior doctors of efficacy and engagement
Source: BMC Med Educ. 2020 Sep 14;20:303. doi: 10.1186/s12909-020-02237-8 (PMC7491171; doi:10.1186/s12909-020-02237-8)
Supplement: Supplementary file 1 — Additional file 1. Perceived Utility of Learning Scale (PULTS). This is an online questionnaire to gather participants quantitative and qualitative perceptions of adaptive tutorials and web-based educational resources in radiology. [file 12909_2020_2237_MOESM1_ESM.docx]

**ADDITIONAL FILE 1 - PERCEIVED UTILITY OF LEARNING SCALE (PULTS)**

*Please indicate to what extent you agree with each of the statements below regarding the educational resource.*

(Likert scale – 1 strongly disagree to 6 strongly agree).

1. I would recommend this learning resource to others.
2. It provided an individualized learning environment.
3. It provided feedback that enhanced my learning.
4. Navigation was simple and straight forward.
5. It met my needs for flexibility in my learning.
6. It made my learning more efficient (saved time).
7. It helped me to identify priorities for my learning.
8. It enhanced by motivation to learn about this topic.
9. It improved by understanding of the topic.

*Please rate your understanding of the topic.*

(Likert scale – 0 least understanding to 10 most understanding)

BEFORE you used the educational resource.

AFTER you used the educational resource.

*Please rate the overall value of the educational resource.*

(Likert scale – 0 not useful to 10 extremely useful).

*Please comment on what you liked most about this educational resource.*

*Please comment on what you would like to see changed in this education resource.*
